# Enhancing Reverse Design Ability of Functional Materials Based on Data Quality Management: Taking Biomedical Zinc Alloy as an Example

**DOI:** 10.3390/ma18204729

**Published:** 2025-10-15

**Authors:** Xujie Gong, Xue Jiang, Shiyu Huang, Yize Wang, Lishen Ding, Yanjing Su, Yu Yan

**Affiliations:** 1 Beijing Advanced Innovation Center for Materials Genome Engineering, Institute for Advanced Materials and Technology, University of Science and Technology Beijing, Beijing 100083, China; d202310739@xs.ustb.edu.cn (X.G.); yjsu@ustb.edu.cn (Y.S.); 2 School of Advanced Materials Innovation, University of Science and Technology Beijing, Beijing 100083, China; jiangxue@ustb.edu.cn; 3 College of Ocean Science and Engineering, Shanghai Maritime University, Shanghai 201306, China; syhuang@shmtu.edu.cn; 4 School of Materials Science and Engineering, University of Science and Technology Beijing, Beijing 100083, China; u202240516@xs.ustb.edu.cn (Y.W.); u202240514@xs.ustb.edu.cn (L.D.)

**Keywords:** data-driven, materials genome engineering, data quality management, zinc alloy

## Abstract

Biodegradable zinc alloys have shown great potential in the biomedical field, but are limited by their poor mechanical properties. Alloying is essential for improving mechanical properties, yet designing multicomponent zinc alloys remains challenging due to complex elemental interactions. Notably, while data-driven active learning approaches offer new strategies for zinc alloy design, data quality issues such as redundancy, outliers, and inconsistencies in multi-source heterogeneous data hinder modeling accuracy and interpretability. In this work, we proposed a data quality management strategy based on recursive screening, targeting three key data problems, namely, redundant data (RD), outlier data (OD), and inconsistent target data (ID). Case studies on hydrogen embrittlement, phase-change refrigeration materials, and matbench_expt_gap datasets showed that, in the aforementioned data-driven research, RD optimized data distribution but risked precision loss in high-performance regions; OD enhanced minority alloy features but risked overfitting; and ID preserved high-performance data, boosting extrapolation but risking underfitting. Six multicomponent zinc alloys were designed and fabricated using these strategies. Experiments showed ID-optimized datasets achieving 482 MPa—near state-of-the-art performance. The highest tensile strength of 482 MPa was obtained in the alloy Zn-1.2Al-0.8Mg-0.45Li-0.3Mn (at%), designed via the ID-optimized dataset. The study revealed that in inverse design, predictive accuracy in high-performance regions outweighs data volume or density, underscoring the value of data quality management for multi-source materials development.

## 1. Introduction

Zinc alloys have emerged as promising candidates for biomedical applications, especially for the development of biodegradable implants such as orthopedic fixation devices, cardiovascular stents, and controlled drug-release systems [1,2,3,4,5]. Unlike conventional Fe and Mg-based biodegradable alloys, zinc alloys provide superior degradation characteristics in physiological environments, exhibiting uniform corrosion behavior at a physiologically appropriate rate [6,7,8]. In addition, zinc’s degradation products positively affect protein composition, bone growth, and anti-arteriosclerosis [9]. However, the clinical implementations of zinc alloys have faced significant challenges due to their suboptimal mechanical performance. Pure zinc has a tensile strength of 100–150 MPa, falling significantly below the minimum requirement of 300 MPa for load-bearing orthopedic applications [10,11,12]. Therefore, alloying is critical to enhancing their mechanical performance. For example, adding Li [13] or Mg [14] can significantly enhance strength, with Mn [14] or Cu [15] ensuring good ductility. These alloying elements not only improve the mechanical properties of biodegradable zinc alloys but also form multiple alloy systems, increasing the design search space and complicating the relationships between elements, composition, microstructure, and mechanical properties.

In recent years, data-driven approaches have provided new avenues for designing multicomponent zinc alloys. Xue et al. [16,17] introduced a materials inverse design strategy based on Bayesian optimization. By training agent models to search within a design space, this method was successfully validated in high-entropy alloys [18], high-temperature superalloys [19], and Cu-based functional materials [20,21].

This approach has been recently extended to biodegradable zinc systems. Guo et al. [22] conducted a preliminary investigation using k-nearest neighbor algorithms to correlate alloying elements with mechanical performance, identifying compressive yield strength as the critical design metric. However, their computational predictions have remained inadequately supported through systematic experimental validation. Chen et al. [23] used ultimate tensile strength (UTS) and immersion corrosion rate as targets to design a 400 MPa grade biodegradable Zn-Mn alloy. These studies confirmed the effectiveness of data-driven methods in designing biodegradable zinc alloys. However, few studies have been conducted on zinc alloys compared with Mg alloys, resulting in more data. Existing biodegradable zinc alloy design research has remained limited to a few systems, resulting in a lack of comprehensive consideration of multiple alloying elements [24,25,26,27]. This can be attributed to research data from different sources, including experiments and published papers, with non-unified testing methods, limited data quantity, and inconsistencies, all of which affect the data-driven design of zinc alloys.

The impact of data quality on data-driven materials research and development has attracted significant attention. Shi et al. [28] proposed a robust data quality governance approach, addressing mismatch between high-dimensional material data feature spaces and small sample sizes, while Liu et al. [29] enhanced model predictability by filtering outlying data. However, prior studies have failed to achieve a clear definition of data quality issues and an in-depth analysis of their impact on the subsequent design of materials.

Redundant data (RD), defined as the repeated storage of identical or superfluous information in databases or data storage systems, often results in overfitting and reduced model generalizability. Li et al. [30] demonstrated that removing RD had a minimal impact on model performance. Rather than focusing solely on data volume, it may be more effective to build smaller, information-rich datasets using uncertainty-based active learning algorithms. Outlier data (OD) are data points that significantly deviate from other observations in a dataset. These can mislead models, preventing them from correctly identifying patterns in the data and reducing their generalizability while distorting statistical analysis results [31]. Wang et al. [32] examined the use of outlier detection indices in high-dimensional molecular dynamics (MD) simulations based on machine learning to identify rare events such as local phase transitions, ion segregation, defect migration, interface reconstruction, and grain boundary sliding. This approach provided a better understanding of material behavior and properties under different conditions. Luis et al. [33,34,35] explored how to detect samples with large expected errors (outliers) using uncertainty quantification in reactive molecular potential energy surfaces to improve the accuracy and robustness of machine learning models. Notably, improving the model’s predictive ability in the high-performance region using a targeted approach also serves as a valid strategy. Zhang et al. [18] improved the accuracy of the model in the high-performance region through feature engineering, enhancing the materials design capability in the active learning process, which also could serve as an important data quality management approach. However, the above-mentioned studies focused extensively on the definition of data quality issues and lacked an analysis of how low-quality data affects materials design.

This study focused on the inverse design of mechanical properties for biodegradable zinc alloys. By employing an active learning optimization method, this approach was used to examine and compare the issues of data redundancy, anomalies, and inconsistencies that emerge from multi-source and heterogeneous datasets commonly found in materials research. The study examined how these factors influenced the accuracy and interpretability of models. Using the design of biodegradable zinc alloys for biomedical applications as a case study, we explored the impact of these three data quality issues within the scope of data-driven inverse materials design. The integration of active learning techniques was used to expedite the design process of biodegradable zinc alloys while still providing robust methodologies for assessing and enhancing data quality in materials development driven by data. There is still a lack of systematic research on how low-quality samples in multi-source heterogeneous data specifically affect the reverse design effect of zinc alloys.

## 2. Methods

### 2.1. Workflow

**Figure 1:**
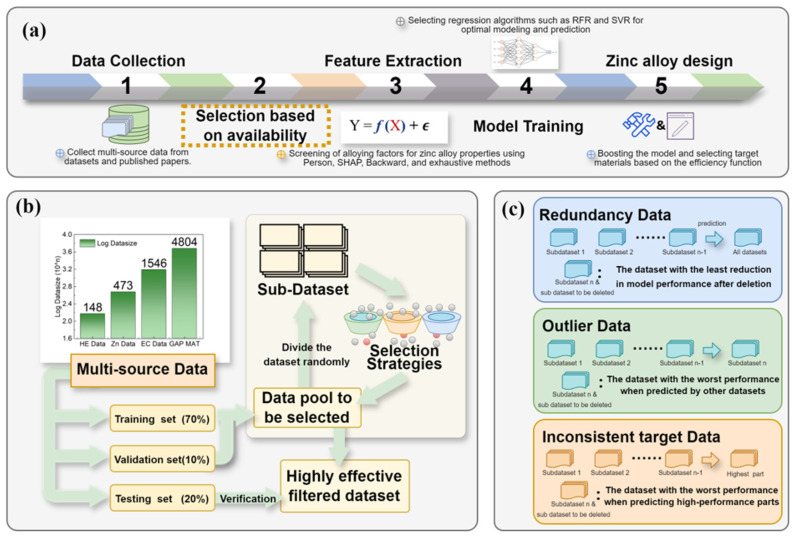
Workflow used in this study: (**a**) proactively learning the process of designing zinc alloys; (**b**) recursive data quality evaluation process; and (**c**) definition of the three data quality issues (RD, OD, and ID).

To efficiently design biodegradable zinc alloys with high tensile strength, in this work, we adopted an active learning workflow, as outlined in Figure 1a. The traditional active learning process for inverse materials design consists of the following key steps: data collection, feature extraction, model building, and Bayesian-optimized inverse design. Given that biodegradable zinc alloys have complex data sources and a multitude of systems, we introduced an additional data quality management step following the data collection phase.

As shown in Figure 1b, data processing was based on iterative recursion. We started by randomly dividing the multi-source data pool into training, validation, and test sets in a 7:1:2 ratio to ensure the diversity and representativeness of the data. It is crucial that the external test set (20% of the entire data) is isolated and only used once in the final evaluation before any recursive cleaning or hyperparameter search. Subsequently, the training set was partitioned into several subsets. Using cross-validation, we screened these subsets for three types of data quality issues: RD, OD, and inconsistent target data (ID). The screening process involved eliminating the subsets that performed the worst until the cross-validation error (MSE) reached twice the initial value. Next, we will use two indicators, R^2^ and MSE, to evaluate the predictive ability of the model. R^2^ measures the proportion of variance explained by the model (values approaching 1 indicate superior fit), while MSE quantifies the average squared deviation between predictions and true values (lower values signify higher accuracy). Together, these complementary metrics provide a balanced assessment of predictive capability, with R^2^ emphasizing explanatory power and MSE highlighting precision.

To ensure the scientific validity and generalizability of our data quality management approach, we used three additional multi-source datasets that varied in size and quality. This study establishes a systematic data quality evaluation framework for data-driven materials design by categorizing issues into three distinct types: Redundant Data (RD)—identified as subsets with repetitive or superfluous information through quantification of minimal model performance degradation upon removal; Outlier Data (OD)—detected as subsets exhibiting significant deviation from the overall distribution via cross-validation errors during training, where the highest error subset is flagged (potentially due to experimental artifacts or unique material mechanisms); and Inconsistent Target Data (ID)—isolated as subsets causing prediction bias in high-performance regions by comparing model accuracy before and after exclusion, with improved accuracy post-removal marking ID. We choose to define the top 10% of performance data as high-performance data in the code. By integrating performance degradation rates, cross-validation errors, and high-performance region prediction accuracy, this framework provides a quantitative, unified approach to assessing data quality in materials informatics. In this work, we proposed three data quality issues (RD, OD, and ID) and designed corresponding recursive screening strategies, as illustrated in Figure 1c.

The above definition can be verified from existing research in information theory and material genetic engineering. RD corresponds to the concept of information redundancy, which identifies duplicate or irrelevant information by quantifying the “minimum model performance degradation” and essentially maximizes the effective information entropy density. In data-driven materials research, Li et al. [30] found that removing RD had limited impact on model performance based on a definition similar to the method proposed in this paper, indicating that compared to simply pursuing data volume, constructing small-scale high information density datasets based on uncertainty active learning may be more effective. OD is based on the information characteristics of “low probability events” with outliers, and uses cross validation error to detect subsets that significantly deviate from the distribution, which conforms to the principle of maximum entropy for reverse application. In data-driven material research, outlier detection has always been one of the key issues in data quality management. Liu et al. [29], Wang et al. [32], Luis et al. [33] have all proposed methods for verifying data outliers, and their core idea is consistent with this paper, which is to construct models based on existing data to determine the reliability of new data. ID identifies the subset of data that causes prediction bias by evaluating the local failure of feature target mutual information in high-performance regions, which is highly consistent with the principle of “preserving the maximum task related information” in information bottleneck theory. It is worth noting that Zhang et al. [18] improved the predictive accuracy of the model in high-performance regions through targeted feature engineering, verifying the value of OD management in active learning material design.

In theory, the number of folds in cross validation is also an important parameter, and the higher the fold, the better the performance. In theory, the LOOCV method has better performance, which means that for zinc alloys (data size *n* = 473), the computational cost will increase by about 50 times. The purpose of this article is to verify the effectiveness of the method, and considering computational efficiency, the most common 10-fold cross validation is chosen for in each round of recursive data quality assessment. As a result, some data without issues were inevitably removed along with problematic subsets. To address this, we repeated the screening process 50 times and calculated the average number of times each data point was screened out, using this metric to evaluate the data quality for each of the aforementioned issues.

### 2.2. Method Validation

**Figure 2:**
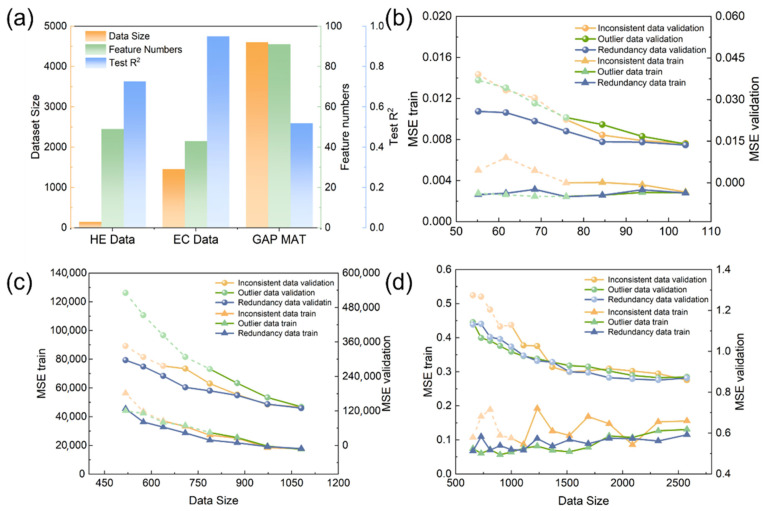
Basic information and data quality management process of the validation dataset: (**a**) validation dataset data volume, feature quantities, and initial modeling accuracy; (**b**) hydrogen embrittlement data (HE); (**c**) phase-change refrigeration materials (EC); and (**d**) matbench_expt_gap (matgap).

As outlined earlier, to showcase the broad applicability of the three data quality management strategies introduced in this work across different materials datasets, we chose three diverse datasets, namely, hydrogen embrittlement stress corrosion data (HE), phase-change refrigeration material data (EC), and matbench_expt_gap (matgap), before conducting the inverse design of mechanical properties for biodegradable zinc alloys. We then performed multiple rounds of recursive data evaluation on these datasets, with their fundamental information and sources detailed in Table 1. Figure 2a presents an overview of the key characteristics of these three datasets, covering the data size, the count of features, and test R^2^ values. The data sizes of the three datasets incremented progressively, and initially, the feature counts were relatively balanced. Among these datasets, the EC dataset exhibited the strongest initial performance, followed by the HE dataset, with the matgap dataset exhibiting the weakest performance.

Figure 2b–d shows how the three strategies affected the model’s training and test MSE across different dataset sizes. To better compare the three data quality management methods, we continued the iterative evaluation even after the OD and ID errors reached the threshold. Instead, we allowed the models to run for the same number of iterations as RD, with extra data indicated by dashed lines. As shown in the figures, MSE generally increased as the screening process continued. This was mainly because larger datasets provided more stable statistical properties. However, the positive and negative impacts of the three methods on the model were clearly observed through comparison.

Among the three methods, RD exhibited the slowest increase in error, requiring more iterations to reach the screening threshold and achieving the lowest error on both the training and validation sets. During iterative data filtering of the three datasets using the three methods, the training set MSE curves consistently showed lower values for OD compared to the other two methods, while its test set MSE was higher—indicating an overfitting risk for OD. Conversely, ID exhibited the poorest performance across all test sets. This method carries the risk of underfitting.

This consistency across the three datasets demonstrated the stability of the RD strategy for these types of problems. In contrast, ID and OD showed the worst performance. ID retained high-performance data by removing the worst-performing subsets during high-performance predictions, thus enhancing the model’s extrapolation ability. As a result, this model performed relatively well on the validation set. However, its excessive focus on high-performance regions led to poor performance on the training set, causing underfitting. OD, however, removed subsets that performed poorly when predicted by other datasets. When these outliers were removed, it also reduced the model’s generalization ability, leading to overfitting.

In summary, RD optimized data distribution and enhanced model generalization. OD also strengthened the feature expression of minority alloy systems but risked overfitting. ID preserved high-performance data and improved model extrapolation but could cause underfitting. These findings provided important insights for selecting appropriate data quality management strategies in materials design.

To more intuitively present the data quality evaluation, we tracked each data point’s average screening status over 50 recursive data filtering rounds and analyzed data quality changes after removing data with an average screening round below 2. The threshold of 2 was chosen based on the training set’s error trend in Figure 2, as most training sets achieved optimal error states after the second iteration.

**Figure 3:**
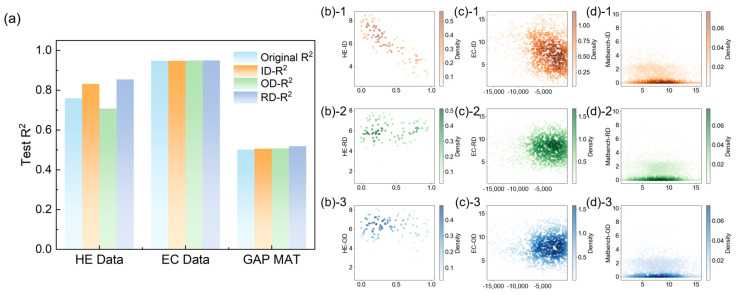
Relationship between the accuracy changes in the test dataset before and after data quality management, average screening epochs of the data, and intrinsic information of the data: (**a**) accuracy changes in the test dataset before and after data quality management (measured as R^2^); (**b**) average number of rounds for screening HE data in ID (**(b)-1**), OD (**(b)-2**), and RD (**(b)-3**) problems; (**c**) average number of rounds for screening EC data in ID (**(c)-1**), OD (**(c)-2**), and RD (**(c)-3**) problems; and (**d**) average number of rounds of screening for matgap data in ID (**(d)-1**), OD (**(d)-2**), and RD (**(d)-3**) problems.

Figure 3a shows how R^2^ changed after partial data removal. For the three datasets, EC initially exhibited the best performance and was least affected by the three data quality strategies. In contrast, the lower the initial quality of the dataset, the more significant the improvement after optimization. Focusing on HE and matgap, ID and RD excelled in enhancing model accuracy. OD, however, had a dual nature, aiding the large matgap dataset but harming the small hydrogen embrittlement dataset due to the removal of some special-patterned data.

This pattern can also be observed in Figure 3b–d, with the *x*-axis showing the target value, the *y*-axis indicating the screening round, and the color intensity indicating data density. The ID method demonstrated a strong linear relationship with the target value, and the poor-performing data were eliminated early. Figure 3b,c, and (d)-2 presents a more concentrated data distribution than Figure 3b,c, and (d)-3 because the outlier screening mechanism removed data that did not fit existing patterns, while the RD strategy focused on reducing high-density data regions. This suggested that the data quality was already poor and that initiating outlier screening possibly removed valid but risky data, making the model conservative and weakening its generalization ability.

In summary, RD optimized data distribution, thereby enhancing model generalization. OD better centralized the data but carried a significant risk of overfitting, while ID helped enhance model generalization.

## 3. Results and Discussion

### 3.1. Degradable Zinc Alloy Dataset

Drawing on our discussion of the HE, EC, and matgap datasets, we obtained 473 entries of mechanical performance data for biodegradable zinc alloys from public sources. This included data from the materials genome engineering database (https://www.mgedata.cn/, accessed on 30 June 2025), results from our previous work [36], and the accumulation of experimental data from relevant studies. The dataset details are shown in Table 2. To accurately explore the impact of data quality on material performance design, we focused on UTS as the core indicator. It is known that severe plastic deformation such as rolling and extrusion can greatly improve the mechanical properties of zinc alloys, so this distinction is necessary. For the process field, we conducted a single heat encoding to distinguish the influence of different processing techniques on zinc alloys. We set features such as rolling and extrusion, and used 1 to represent the material using this process. Due to the general lack of record in the temperature field of zinc alloy processing technology in existing datasets, we did not adopt the feature of processing temperature in our modeling process.

**Figure 4:**
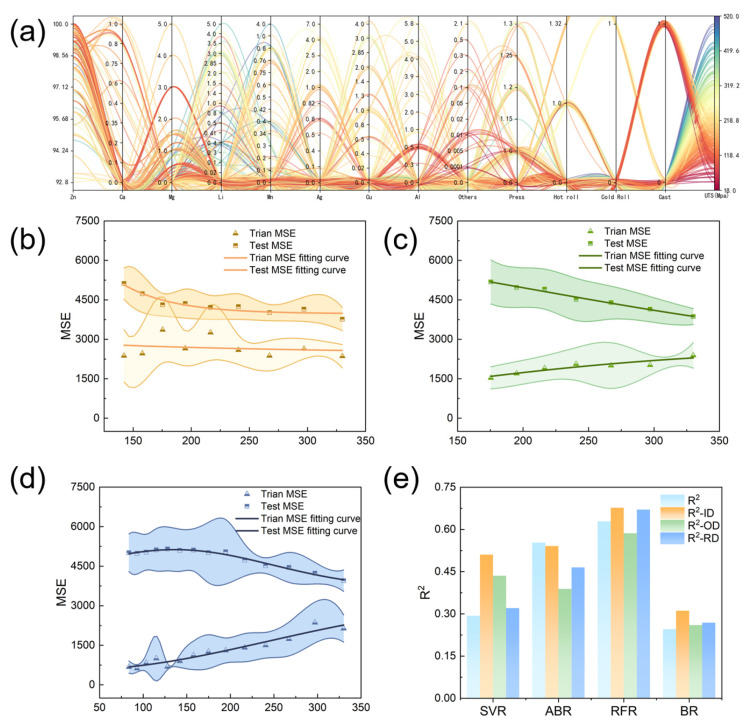
Characteristics distribution and data quality management results of the degradable zinc alloy dataset: (**a**) parallel diagram between the degradable zinc alloy features; (**b**) iterative evaluation of error changes in the degradable zinc alloy dataset using the ID strategy; (**c**) OD strategy to iteratively evaluate the error variation in the degradable zinc alloy dataset; (**d**) RD strategy to iteratively evaluate the error variation in the degradable zinc alloy dataset; and (**e**) model errors before and after data quality management.

Figure 4a displays the combined impact of alloying elements and process components on UTS within the merged dataset. Most biodegradable zinc alloys clustered around 200 MPa, with a few exceeding 500 MPa. We then applied three recursive data quality assessment strategies, namely, ID, OD, and RD, to this dataset, running 50 trials for each to calculate the average dataset errors and screening rounds. The results indicated that RD, with the most screening rounds, most effectively reduced errors and optimized data distribution. However, each strategy had distinct advantages and limitations.

ID initially slightly improved testing set performance but later caused the training set errors to rise and then fall. This was because ID retained more high-performance data, enhancing the model’s extrapolation ability in this area but causing underfitting. Meanwhile, OD caused a continuous decrease in training set errors but an increase in testing set errors, resulting in overfitting. While removing outliers, OD possibly eliminated critical data, harming the model’s generalization. Finally, RD caused significant initial error increases, but these stabilized as screening progressed, resulting in smaller error increases than those caused by ID and OD.

After filtering data with an average screening round >2, we used four machine learning methods for prediction. The managed datasets showed slightly worse performance in adaptive boosting regression (ABR) but significantly better support vector regression (SVR) and Bayesian regression (BR) performance, proving the effectiveness of data quality management. Random forest regression (RFR) showed the highest prediction accuracy, while RD and ID performed well, and OD experienced overfitting, consistent with prior analyses.

### 3.2. Analysis and Discussion

**Figure 5:**
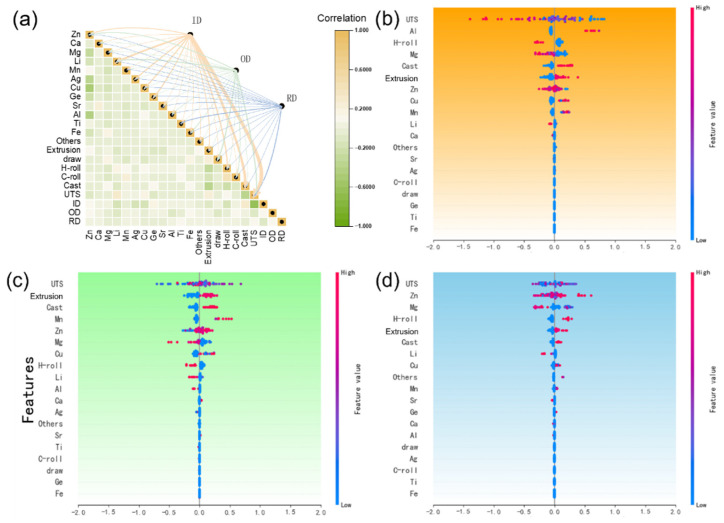
Relationship between the average screening rounds and features of the degradable zinc alloy data: (**a**) heat map of the correlation between the screening rounds and features under three data quality management methods for degradable zinc alloys; (**b**) SHAP relationship between the average screening rounds and features under the ID method; (**c**) SHAP relationship between average screening rounds and features under the OD method; and (**d**) SHAP relationship between average screening rounds and features under the RD method.

To clarify the impact of the three data quality management strategies (ID, OD, and RD) on the zinc alloy dataset and the inverse design of new biodegradable alloys, we examined their relationships with the features of biodegradable zinc alloys. Figure 5a displays a Pearson correlation heatmap quantifying the relationships between three data-quality strategies (ID, OD, RD) and representative zinc-alloy features (e.g., Al, Li, Mn, and processing parameters), with color intensity indicating the strength of each association. The ID strategy exhibits strong positive correlations (>0.6) with key features such as Al and Li, demonstrating that retaining high-performance data markedly improves the prediction accuracy of high-strength alloys. In contrast, the OD strategy shows elevated correlations with elements such as Ti and Sr but with reversed signs, revealing that outlier removal enhances the representation of minority alloy systems while potentially impairing model generalization. The RD strategy, characterized by weaker correlations with most features, reflects its focus on balancing data distribution to optimize overall model robustness, albeit without targeted enhancement of high-performance regions.

Figure 5b–d illustrates the relationship between feature variables and screening rounds. Features such as Al, Li, and extrusion had longer retention rounds, indicating their importance in predicting high-performance regions and enhancing the model’s performance in this area. The OD method pays more attention to whether this data belongs to a certain minority system, and thus identifies it as an outlier in advance. From Figure 5c, it can be seen that compared to the two methods, it pays less attention to the main elements such as Zn and Li, and more attention to elements such as Ti and Ag, especially Ag. As shown in Table 2, the vast majority of the data does not contain this element. The OD method focuses more on whether the sample contains this minority element and tends to label it as an outlier. This aligned with prior analysis, where OD enhanced the feature expression of some alloy systems, possibly reducing data diversity and increasing overfitting risks. The RD algorithm, which removed high-density data regions to optimize distribution and improve model generalization, had a complex impact on high-performance regions, warranting further study. Unlike ID and OD, RD focused on overall data distribution optimization.

### 3.3. Material Reverse Design

This study first constructed three optimized zinc alloy datasets based on three data quality management strategies: redundant data (RD), abnormal data (OD), and inconsistent target data (ID). Each dataset was trained using the Random Forest Regression (RFR) algorithm to train independent surrogate models, with RD model R^2^ = 0.85, OD model R^2^ = 0.82, and ID model R^2^ = 0.87, all significantly better than the baseline model of the untreated dataset (R^2^ = 0.78). Through 10-fold cross-validation and Bayesian hyperparameter optimization, the average absolute error (MAE) of the three models on the test set was controlled within 15 MPa, providing a reliable predictive basis for subsequent reverse design. The differences in R^2^ among the three strategies are consistent with the aforementioned data bias: retaining high-performance samples for ID and improving the signal-to-noise ratio in high-value areas, with R^2^ reaching 0.87; OD eliminates rare cases, distorts the training set, and reduces R^2^ to 0.82; RD only removes duplicates, with balanced information density and a median R^2^ of 0.85. The increase in relative uncleaned data (+0.09~+0.11) confirms that targeted cleaning is more effective in improving model interpretability than simply scaling.

In response to the complexity of the zinc alloy system with numerous elements, this study focuses on the four key alloy elements with the highest proportion of non-zero elements: Mg (0–1%) (including the left endpoint but not the right endpoint, for a total of 40 levels, the same applies below), Li (0–0.8%), Mn (0–1%), Al (0–2%), Construct a discrete search space with a step size of 0.05%. Due to the need for severe deformation to improve the mechanical properties of zinc alloys, we have uniformly set the processing technology to extrusion to reduce the search space. The final design space consists of 256,000 candidate alloys. This strategy ensures the diversity of element combinations and provides search space for the next step of material screening.

Based on the three trained proxy models, this study adopts the upper confidence interval (*UCB*) method for multi-objective optimization screening. The UCB function is defined as:

$$UCB\left(x\right)=\mu \left(x\right)+\sigma (x)$$

where *µ*(*x*) is the predicted UTS mean and *σ*(*x*) is the uncertainty. After independently calculating the UCB value for each model, the weighted average of the three was taken and sorted. Finally, two high-potential alloys were selected from each model for experimental verification. The results are shown in Table 3. It should be emphasized that the RD/OD/ID quality management strategy proposed in this article is currently only applicable to the single objective of “room temperature tensile strength”. If multidimensional indicators such as ductility, corrosion rate, and cell compatibility are introduced simultaneously, not only will the data distribution sparsity and dimensional differences in each indicator be significantly amplified, but recursive filtering under cross validation will also lead to an exponential decrease in the available sample size, thereby weakening the trainability and interpretability of the model. Therefore, it is not realistic to verify the feasibility of our method in all dimensions at once. The follow-up work will expand the dataset in stages and explore the transferability and scalability of data quality strategies within a multi-objective Bayesian optimization framework.

### 3.4. Experimental Verification

Using the aforementioned workflow, we designed six biodegradable zinc alloys, whose compositions are listed in Table 4. Al, Mg, Li and Mn jointly retard recrystallization and grain growth, preserving a fine as-extruded structure that underpins the model-predicted high strength; prolonged post-extrusion holding would trigger slight softening, but immediate quenching was applied to maintain the designed state. To produce these alloys, we prepared high-purity raw materials according to the table and employed vacuum arc melting. After verifying and adjusting the vacuum arc melting furnace, the materials were loaded, the chamber was evacuated to 10^−2^ Pa, and melting was initiated for 30 min. We then flipped the furnace two to three times to ensure compositional uniformity and finally cast the molten metal into ingots. Subsequently, extrusion processing was performed on the zinc alloy ingots. The ingots were machined into ϕ30 mm billets and immediately hot-extruded at 320 °C to ϕ15 mm, corresponding to an extrusion ratio of ~4 (true strain ≈ 1.4). This degree of deformation was selected on the basis of our previous systematic study [37], in which an ~80% reduction at 320 °C was found to produce the finest dynamically recrystallised grain structure and the optimum strength–ductility balance in Zn–(Al, Mg, Li, Mn) alloys. After extrusion, the rods were sectioned and machined into flat tensile specimens with a gauge length of 25 mm, width ~5 mm and thickness ~1.1 mm, following GB/T 228.1-2021 [38].

**Figure 6:**
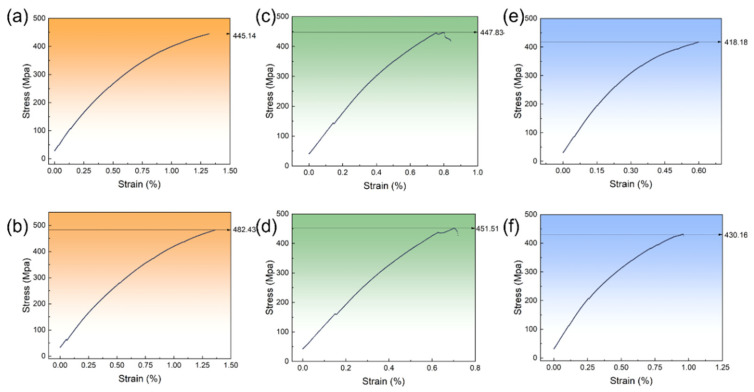
Degradable zinc alloy composition tensile curves designed by the proxy model, following processing using different data quality evaluation methods: (**a**,**b**) ID; (**c**,**d**) OD; and (**e**,**f**) RD.

As shown in Figure 6, we designed two material groups for each of the three data quality management strategies, resulting in a total of six material groups. The tensile tests were conducted at 25 °C on a Zwick/Roell Z050 testing machine (ZwickRoell GmbH & Co. KG, Ulm, Germany), following GB/T 228.1-2021 [38]. Plate specimens with 25 mm gauge length, ~5 mm width, and ~1.1 mm thickness were used. A 25 mm extensometer was applied, and the strain rate was 10^−3^ s^−1^. The detailed repeated data and error results of the tensile test have been shown in Table 5. The average UTS of these materials ranged from 418 to 482 MPa, aligning with previous studies demonstrating the model’s effectiveness in inverse design. A comparison of the three strategies revealed average UTS values of 471 MPa (ID), 449.5 MPa (OD), and 422 MPa (RD). Notably, RD, while exhibiting the best performance in overall model evaluation, delivered the worst practical design results.

**Figure 7:**
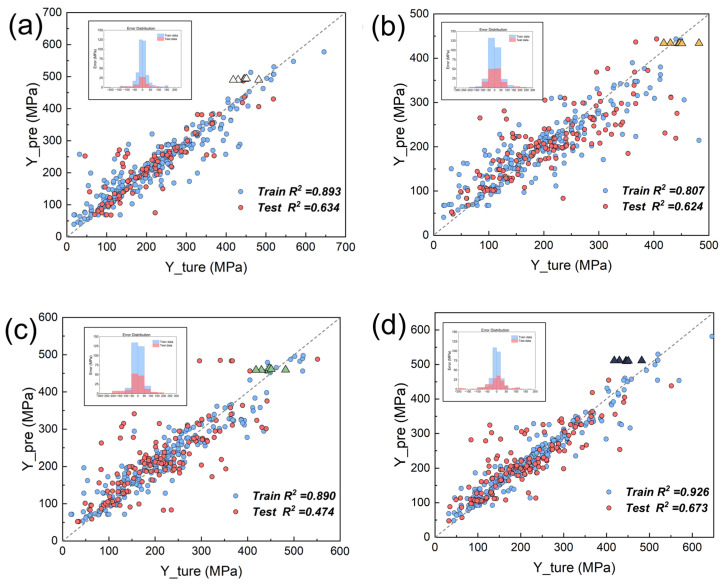
Accuracy of modeling training sets, testing sets, and new testing areas before and after different data quality management methods: (**a**) original dataset; (**b**) ID; (**c**) OD; and (**d**) RD.

To explore this discrepancy, Figure 7 compares the prediction-actual value plots of the managed datasets with the unmanaged dataset. These triangles represent the newly conducted experiment. We calculated the MAE predicted by different data quality management methods for the high-performance data area (all above 400 MPa) obtained from our experiment. The original model had a MAE of 45.66 MPa for this area, while the ID was 18.46 MPa and the OD was 21.93 MPa. However, the MAE of the RD method was as high as 65.09 MPa. The ID-based model showed slightly worse fitting, even underfitting, with no improvement on the test set and reduced predictive power on the training set. However, it better predicted the performance of the six new materials. Similarly, the OD-based model displayed the same pattern. When all data were used for training, the RD-screened dataset experienced overfitting and poor performance on the test set. This was because RD enhanced the mainstream data in the high-quality dataset but underfit the high-performance region. Thus, RD improved the model’s overall capability but not its high-performance, design-focused ability.

## 4. Conclusions

This study investigated the active learning design process of degradable zinc alloy tensile strength and systematically analyzed the impact of multi-source heterogeneous data quality issues on model performance, thereby improving the efficiency and accuracy of material reverse design. This study proposed a data quality strategy based on recursive filtering and defined three data quality issues: RD, abnormal data, and inconsistent target numbers. The design of degradable zinc alloys was validated using three validation datasets, namely, hydrogen embrittlement, phase-change refrigeration materials, and matbench. The results showed that RD not only optimized the data distribution and significantly improved model generalization ability but also potentially weakened the prediction accuracy of high-performance regions. Meanwhile, OD enhanced the characteristic expression of a few alloy systems; however, there was a risk of overfitting. Prioritizing the retention of high-performance region data for ID enhanced the model’s extrapolation ability on target performance but possibly led to underfitting. By combining the recursive data filtering strategy with an active learning framework, the reverse design model constructed based on the filtered dataset performed well in four machine learning algorithms (ABR, SVR, BR, and RFR), especially in the RFR where the prediction accuracy of the test set was significantly improved. Experimental verification showed that analyzing the impact of three types of data quality management on materials design revealed that although ID and OD strategies resulted in lower predictive performance of the dataset, they showed better design capabilities for degradable zinc alloys due to their improved predictive ability in the high-performance stage. Among these, the zinc alloy designed on the ID-optimized dataset had an average tensile strength of 482 MPa, which was close to the forefront level of existing high-performance zinc alloys. The above results clearly demonstrated that in the reverse design of materials, compared with the predictive ability of materials in high-performance regions, data volume or information density were not the key factors in the mechanical property design of degradable zinc alloys. In addition, we verified the positive effect of data quality management on materials design, demonstrating that improving the prediction accuracy of high-performance regions is more critical than simply increasing data volume or density. The framework proposed in this study provides a reusable solution for the multi-source, data-driven development of materials while also providing a theoretical basis for data quality assessment in materials design.

## Figures and Tables

**Table 1:** Source and basic information of the dataset used for verification.

Dataset Name	Target	Data Size	Data Sources
Hydrogen embrittlement dataset	Hydrogen-induced plasticity loss	148	http://mged.nmdms.ustb.edu.cn/task/#/ (accessed on 30 June 2025)
Phase-change refrigeration material (electrostriction)	Electrostriction	473	http://223.223.185.189:3010/#/ (accessed on 30 June 2025)
Matbench_expt_gap dataset	Gap expt	4804	https://matbench.materialsproject.org/ (accessed on 30 June 2025)

**Table 2:** Basic dataset details of the degradable zinc alloy.

Feature Name	Max. Value	Min. Value	Variance	Non-Zero Ratio
Zn	100 at%	93 at%	1.3559	100%
Ca	1 at%	0	0.1840	9.7%
Mg	4 at%	0	0.7052	42.7%
Li	0.8 at%	0	0.1753	17.5%
Mn	1 at%	0	0.2293	14.6%
Ag	7 at%	0	0.7223	6.5%
Cu	4 at%	0	0.8416	10.3%
Ge	5 at%	0	0.4714	2.3%
Ti	1 at%	0	0.0877	8.2%
Sr	1.1 at%	0	0.1990	6.1%
Al	5.8 at%	0	0.7244	7.8%
UTS	513 MPa	18 MPa	12,289.03	100%

**Table 3:** Performance comparison on the unified test set and high-strength subset (MAE in MPa).

Dataset	${MAE}_{SVR}$	$R_{SVR}^{2}$	${MAE}_{ABR}$	$R_{ABR}^{2}$	${MAE}_{RFR}$	$R_{RFR}^{2}$	${MAE}_{BR}$	$R_{BR}^{2}$
**ID**	63.88	0.293	60.02	0.554	52.47	0.586	72.78	0.246
**OD**	51.51	0.511	51.49	0.541	38.37	0.677	63.07	0.311
**RD**	55.47	0.436	57.98	0.389	41.23	0.628	66.24	0.260
**Baseline**	58.49	0.321	50.84	0.465	38.03	0.670	65.82	0.269

**Table 4:** Degradable zinc alloy composition designed by agent model.

ID Number	Zn (at%)	Al (at%)	Mg (at%)	Li (at%)	Mn (at%)
ID-1	97.5	1.2	0.6	0.4	0.3
ID-2	97.25	1.2	0.8	0.45	0.3
OD-1	98.65	0	0.6	0.45	0.3
OD-2	98.5	0	0.8	0.4	0.3
RD-1	97.9	0.8	0.6	0.4	0.3
RD-2	97.8	0.8	0.6	0.4	0.4

**Table 5:** Degradable zinc alloy repeated tensile test and its results.

Sample Number	Zn (at%)	Al (at%)	Mg (at%)	Li (at%)	Mn (at%)	UTS (MPa)
ID-1-1	97.5	1.2	0.6	0.4	0.3	445
ID-1-2	97.5	1.2	0.6	0.4	0.3	477
ID-2-1	97.25	1.2	0.8	0.45	0.3	482
ID-2-2	97.25	1.2	0.8	0.45	0.3	480
OD-1-1	98.65	0	0.6	0.45	0.3	447
OD-1-2	98.65	0	0.6	0.45	0.3	459
OD-2-1	98.5	0	0.8	0.4	0.3	451
OD-2-2	98.5	0	0.8	0.4	0.3	441
RD-1-1	97.9	0.8	0.6	0.4	0.3	418
RD-1-2	97.9	0.8	0.6	0.4	0.3	415
RD-2-1	97.8	0.8	0.6	0.4	0.4	430
RD-2-2	97.8	0.8	0.6	0.4	0.4	425

## Data Availability

The publicly available data used in this study has been presented in the article table, while the remaining data is subject to review http://223.223.185.189:3010/#/ (accessed on 30 June 2025)Database support. The original machine learning data presented in the study are openly available in http://mged.nmdms.ustb.edu.cn/task/#/ (accessed on 30 June 2025), http://223.223.185.189:3010/#/ (accessed on 30 June 2025) and https://matbench.materialsproject.org/ (accessed on 30 June 2025). The experiment data presented in this study are available on request from the corresponding author due to We are conducting further research based on this data.
